# Engineering Sensory Ganglion Multicellular System to Model Tissue Nerve Ingrowth

**DOI:** 10.1002/advs.202308478

**Published:** 2023-12-19

**Authors:** Junxuan Ma, Janick Eglauf, Sibylle Grad, Mauro Alini, Tiziano Serra

**Affiliations:** ^1^ AO Research Institute Clavadelerstrasse 8 Davos 7270 Switzerland; ^2^ ETH Zürich Rämistrasse 101 Zürich 8092 Switzerland; ^3^ Complex Tissue Regeneration Department MERLN Institute for Technology‐Inspired Regenerative Medicine Maastricht University Universiteitssingel 40 Maastricht 6229ET Netherlands

**Keywords:** acoustic assembly, advanced in vitro models, alternatives to animal testing, multicellular system, sensory nerve ingrowth

## Abstract

Discogenic pain is associated with deep nerve ingrowth in annulus fibrosus tissue (AF) of intervertebral disc (IVD). To model AF nerve ingrowth, primary bovine dorsal root ganglion (DRG) micro‐scale tissue units are spatially organised around an AF explant by mild hydrodynamic forces within a collagen matrix. This results in a densely packed multicellular system mimicking the native DRG tissue morphology and a controlled AF‐neuron distance. Such a multicellular organisation is essential to evolve populational‐level cellular functions and in vivo‐like morphologies. Pro‐inflammatory cytokine‐primed AF demonstrates its neurotrophic and neurotropic effects on nociceptor axons. Both effects are dependent on the AF‐neuron distance underpinning the role of recapitulating inter‐tissue/organ anatomical proximity when investigating their crosstalk. This is the first in vitro model studying AF nerve ingrowth by engineering mature and large animal tissues in a morphologically and physiologically relevant environment. The new approach can be used to biofabricate multi‐tissue/organ models for untangling pathophysiological conditions and develop novel therapies.

## Graphical abstract

This is the first in vitro annulus fibrosus (AF) nerve ingrowth model where the AF's neurotrophic and neurotropic effects are demonstrated in a mature large animal dorsal root ganglion (DRG) system. Acoustic generated mild hydrodynamic forces orchestrate physiological‐relevant multicellular DRG morphology and anatomical‐relevant AF‐DRG distance, which determines their evolved functions.

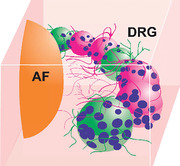

## 1 Introduction

Sensory nerve ingrowth has been observed in the recovery from injury and in many degenerative diseases. In the healing of bone^[^ ^1^ ^]^ and tendon,^[^ ^2^ ^]^ sensory nerve ingrowth into the regenerating tissue may actively participate in tissue repair. On the contrary, aberrant innervations in joints,^[^ ^3^ ^]^ intervertebral disc (IVD),^[^ ^4^ ^]^ and non‐healed bone^[^ ^5^ ^]^ are frequently associated with chronic musculoskeletal pain.

The IVD is a fibrocartilaginous spacer in the spine. The nerve structures in human IVD have been well characterized in former studies^[^ ^6^ ^]^ using calcitonin gene‐related peptide (CGRP) and substance P (SP) to label the axons of nociceptors (i.e., the damage sensing and pain initiating neurons^[^ ^7^ ^]^). They found that in normal, healthy IVD, nociceptor axons are localized only to the surface (< 0.5 mm) of the annulus fibrosus (AF, the fibrous tissue in the outer region of IVD).^[^ ^6a^ ^]^ In contrast, in patients with back pain, nociceptor axons penetrate throughout the entire layer of the AF.^[^ ^6b^ ^]^ These axons in the painful IVD express growth‐associated protein 43, indicating that they are actively growing.^[^ ^6c^ ^]^

Cell bodies of nociceptors locate in the dorsal root ganglion (DRG), an oval tissue within the spinal intervertebral foramina proximal to the IVD.^[^ ^8^ ^]^ Despite the clear association between sensory nerve ingrowth and discogenic pain, little is known about the biological role and mechanism of the AF nerve ingrowth. This points to an unmet need of experimental models. Currently, only in vivo rodent models have been reported to characterize sensory nerve ingrowth in IVD.^[^ ^9^ ^]^ However, the experimental manipulation and visualization of the tiny and complex axonal structures in vivo is challenging. Additionally, many rodent models are poor in predicting human diseases. More than 85% of their predicted therapeutic agents failed in human clinical trials.^[^ ^10^ ^]^ Mice and human have a largely divergent gene expression profile and regulatory program, although they do share the same set of genes.^[^ ^11^ ^]^ It has been generally recommended that, besides rodent models, therapeutic agents should be tested in large animal models before clinical trials.^[^ ^12^ ^]^ Nevertheless, large animal models are expensive, have low throughputs, and raise ethical concerns. Recently, human induced pluripotent stem cell (iPSC)‐derived organoids are developed to faithfully mimic functional human organs but are still limited in studying multi‐organ communication (e.g., between nerve and target tissue).^[^ ^13^ ^]^ The stepwise differentiation protocol to develop human neural organoid takes months. Moreover, the complexity of the nervous system containing various cell types (different types of neurons, glial and vessel cells *etc*.) is not yet fully recapitulated.^[^ ^14^ ^]^

Currently, to the best of our knowledge, there is no in vitro model to study the AF sensory nerve ingrowth. The challenge is to culture target tissue (i.e., AF) and DRG in a physiologically and morphologically relevant condition. First, DRG neurons surrounded by satellite glial cell (SGC) envelope^[^ ^8^ ^]^ are densely packed in a multicellular architecture.^[^ ^15^ ^]^ This close spatial relationship is essential for their physiological crosstalk.^[^ ^16^ ^]^ Secondly, the model needs to recapitulate the close anatomical proximity between DRG and target tissue. The distance between nerve and target tissue may influence their communication.

Modern bioprinting allows microscale‐precise positioning of cells/tissues where cells are usually sparsely distributed in a biocompatible hydrogel‐based ink.^[^ ^17^ ^]^ During bioprinting, cells are subjected to a variety of stimuli that can result in cell damage or death.^[^ ^18^ ^]^ Importantly, DRG neurons’ role is to sense environmental stimuli.^[^ ^19^ ^]^ Therefore, they may be ill‐suited for the common bioprinting processes. Recently, acoustic bioassemblies emerge as rapid, contactless and mild strategies to biofabricate physiologically relevant tissues.^[^ ^20^ ^]^ These techniques coordinate the assembly of biological components through the emergence of specific fluid patterns (i.e., pressure fields, surface instabilities/waves) upon excitatory external stimuli. The precise control of the external stimuli (i.e., frequency and amplitude of chamber vibration) generate hydrodynamic forces that orchestrate the assembly of biological components in predetermined morphologies.^[^ ^20^ ^]^ Once assembled, biological components are in tight cell‐to‐cell contact, dominant requirement to translate from morphed tissues‐like structures to functional tissues!^[^ ^13b^ ^]^

**Figure 1:**
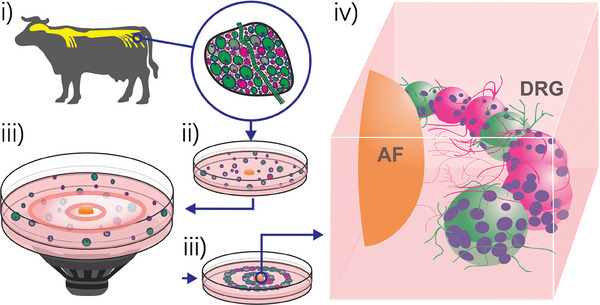
Schematic representation of the experimental approach used to engineer the sensory ganglion multicellular system. Bovine DRG i) is disintegrated into micro‐scale tissue units ii) and assembled iii) in ring‐shaped multicellular systems surrounding the annulus fibrosus (AF) explant iv).

We use Faraday waves^[^ ^21^ ^]^ to assemble large animal DRG micro‐scale tissue units into densely packed architecture to which we refer to as multicellular system (**Figure** **1**). The resultant DRG multicellular system is positioned in the predetermined ring‐shaped geometry surrounding the AF tissue explant in a collagen matrix. We demonstrate the functional intercellular crosstalk and the structural self‐organisation evolved in the cultured DRG multicellular system.

The tissue nerve ingrowth is steered by two key mechanisms. The first is the neurotrophic (growth‐promoting) effect which takes the role of elongating axons. Nevertheless, a simple elongation is not enough without a correct growing direction. Hence, the neurotropic (guidance) effect is of equal importance.^[^ ^22^ ^]^ We evaluated the influence of AF on these two mechanisms of axonal growth in the multicellular system as a proof of concept that pathophysiological nerve ingrowth can be engineered in vitro.

## 2 Results

### 2.1 Fabrication of DRG Multicellular System Surrounding the AF Explant

**Figure 2:**
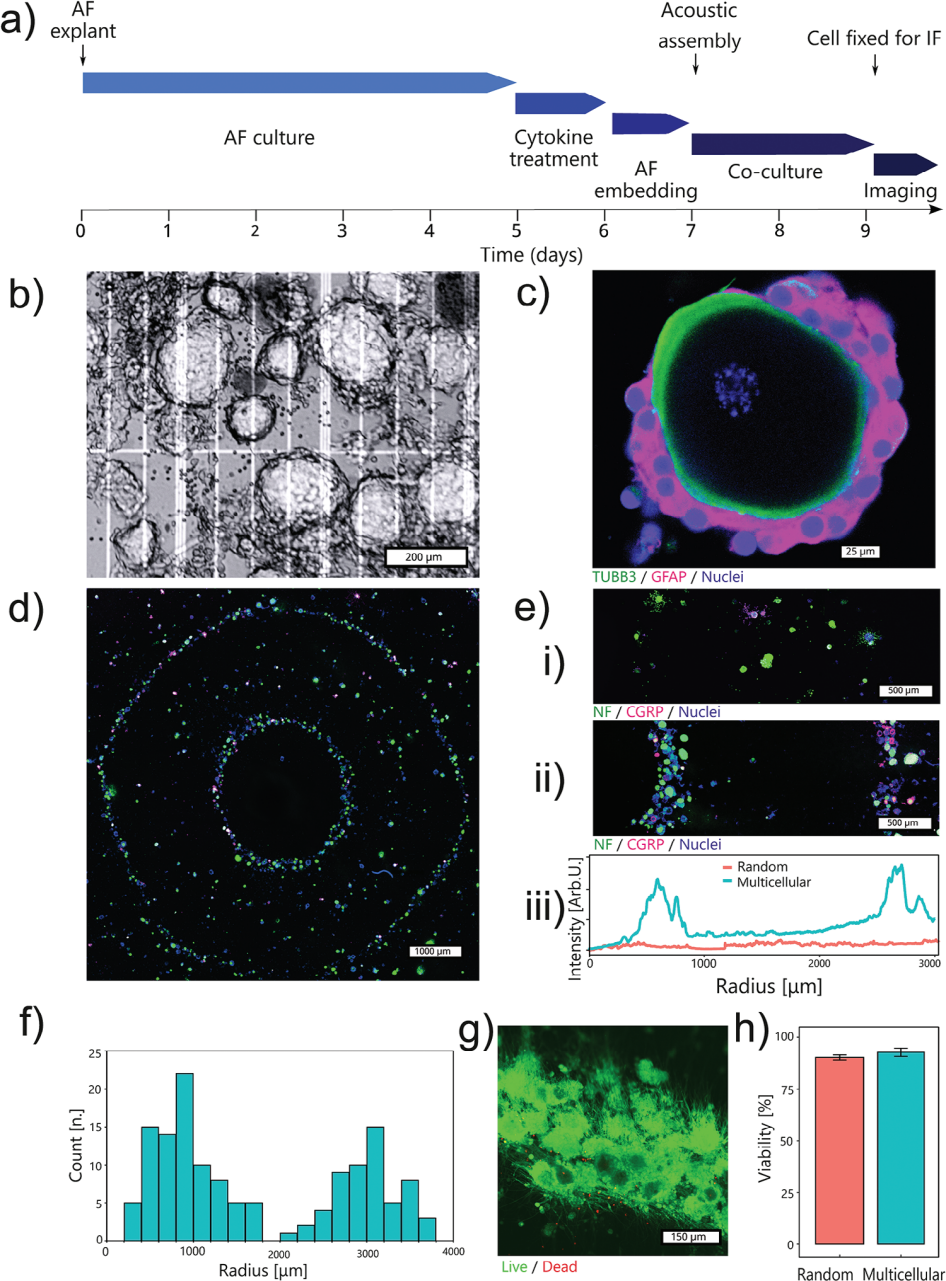
Establishment of the in vitro model to study the AF nerve ingrowth. a) Experimental timeline. b) Phase contrast image of enzymatically dissociated bovine DRG micro‐scale tissue units (trypan blue staining). c) The mild enzymatic procedure preserves the envelope structure of native DRG tissue where neurons stained by tubulin beta 3 class III (TUBB3) are attached by satellite glial cells stained by glial fibrillary acidic protein (GFAP). d,e) Hydrodynamic forces assemble DRG micro‐scale tissue units from a random spatial distribution (i) to a well‐defined geometry (ii and iii). f) This assembly process leads to defined neurons‐AF distances. g,h) The assembly procedure is mild. Viability of the assembled multicellular structure remains above 90% after 2 days of culture. Viability data is presented as mean ± standard error in the bar plot. *n* = 3 replicates per group.

The timeline of AF nerve ingrowth model fabrication is depicted in **Figure** **2**a. The fabrication process starts with a pro‐inflammatory cytokine priming of AF tissue explant to mimic the discogenic pain microenvironment. The applied cytokines are interleukin‐1beta (IL‐1β) and tumor necrosis factor alpha (TNF‐α). These are well known to be associated with clinical discogenic pain.^[^ ^23^ ^]^ The cytokine‐primed bovine AF explant cultured *ex vivo* shows high viability (Figure S1, Supporting Information).

We enzymatically disintegrate the whole DRG explant into micro‐scale tissue units with high viability (87.1% by trypan blue staining, averaged from 24 independent experiments) (Figure 2b). Importantly, the products of enzymatical disintegration are not single cells but tissue units. The satellite glial cells (SGCs) “envelopes” surrounding the neurons are preserved in the tissue units (Figure 2c). This structure is known to underly the neuron‐glia communication.^[^ ^16b^ ^]^

To fabricate the AF‐DRG system, the AF explant is placed in the center of a culture frame with the DRG tissue units dispensed around. This is followed by assembling the DRG tissue units into ring‐shape geometry using hydrodynamic drag forces generated by a vertical vibration (Figure 2d). The ring diameters that determine the AF‐DRG distance (Figure 2e (ii,iii)) are consistent among different independent experiments (Figure S2, Supporting Information). Seeding DRG tissue units without applying hydrodynamic forces leads to their random positioning (Figure 2e (i)). Neurons in the smallest ring are defined as “AF‐close” neurons. The “AF‐close” neurons are enriched at 0.8 ± 0.4 mm from the AF boarder. Neurons in the second ring serve as an “AF‐far” control and their distance to the AF boarder is 3.2 ± 0.8 mm (Figure 2f). The mild assembling process does preserve cell viability. Live / dead staining after 2 days of culture exhibits 92.8% viability in the assembled multicellular system which is at the same level as the random culture (90.3%) (Figure 2g,h).

### 2.2 Functional Characterisation of the DRG Multicellular System

**Figure 3:**
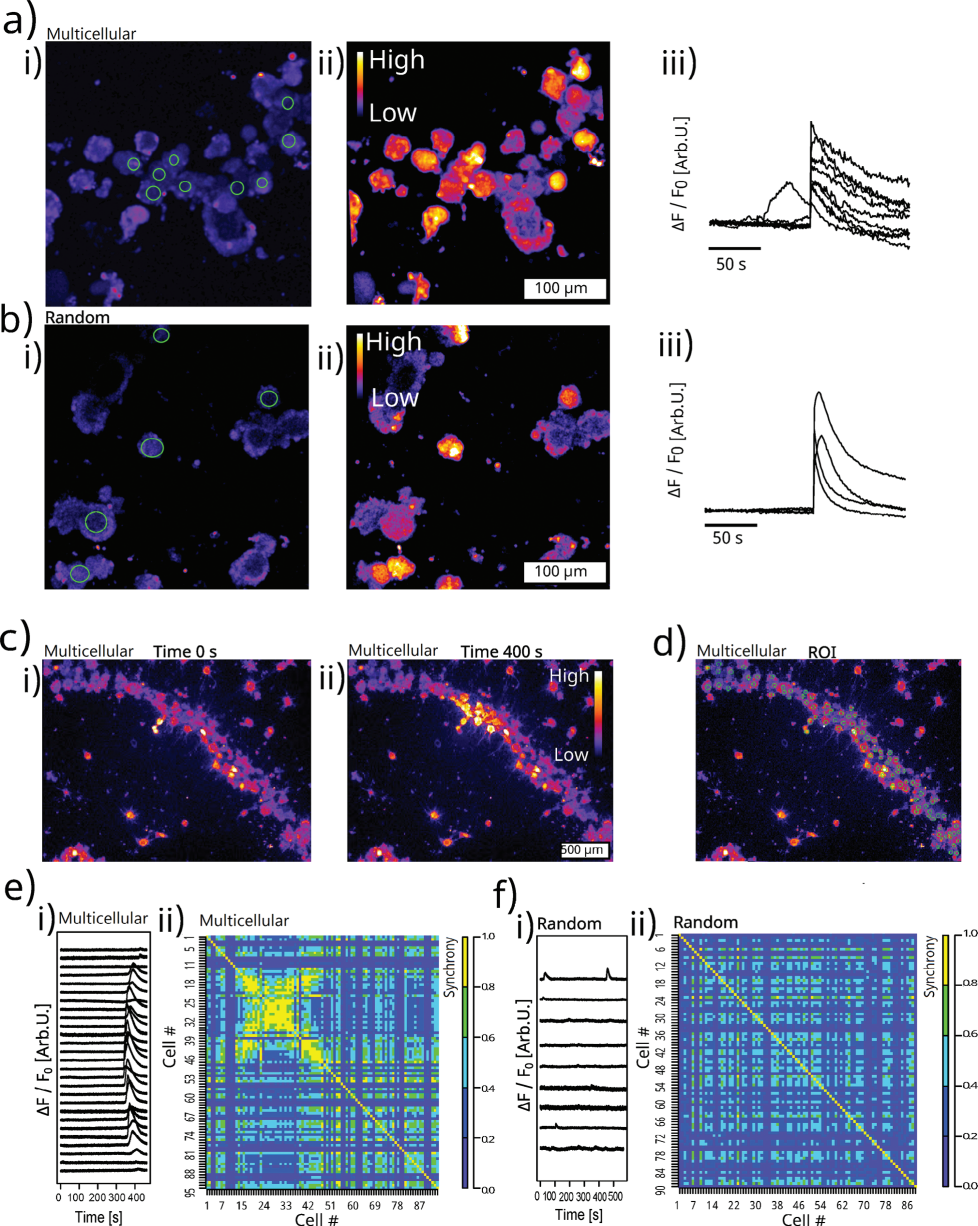
Calcium signal evaluation. a,b) Calcium imaging shows that neurons are functional when depolarised by 50 mM potassium chloride at day 2. c) Neurons in the multicellular system evolve a functional crosstalk after 4 days of culture. The calcium signal is transmitted from one cell to its neighbours only in the multicellular system. The colour scale of a–c) are ΔF / F_0_. d) The region of interest (ROI) of the tissue units. e,f) The multicellular system (e) displays higher synchrony of calcium signal in the tissue units than in its random counterpart f). (i) in (e,f): Normalised calcium fluorescent curves. Only tissue units with spontaneous calcium signals are presented. (ii) in (e,f): The synchrony matrix.

Potassium‐induced depolarization is commonly used to evaluate the functional maturation of neurons in a developing neural organoid.^[^ ^24^ ^]^ Rising the extracellular potassium chloride concentration to 50 mm causes a 48‐mV membrane depolarization in neurons. This leads to the calcium influx through L‐type voltage gated calcium channels (VSCCs) that can be detected through calcium imaging.^[^ ^24b^ ^]^ The maturity of neurons can be evaluated by this potassium depolarization‐caused calcium influx. The functionality of several neural organoids has been evaluated following this method suggesting that 18–30 days of differentiation are needed for neurons to become functionally mature.^[^ ^24^, ^25^ ^]^ In our model, the DRG tissues are obtained from adult cattle and the DRG neurons are readily functionally mature. Indeed, fast calcium upsurge in neurons following potassium depolarization is observed in both random culture and the multicellular system at day 2 (**Figure** **3**a,b). This proves that they are functionally mature without the need of long‐term differentiation.

Despite the activation of individual neuron, the cellular function at the population level is largely unveiled. in vivo DRG exhibits functional crosstalk within the network of neurons and SGCs. The calcium signals can transmit from cell to cell involving both SCGs and neurons.^[^ ^16a^ ^]^ Our multicellular system of DRG recapitulates this in vivo functional crosstalk. The calcium signals travel over a distance of 200–500 µm, crossing 5–20 tissue units (Figure 3c; Video S1, Supporting Information). When the active fluorescent traces are extracted from regions of interest (ROIs) of the DRG tissue units, the spikes of calcium signals are exclusively synchronized in the multicellular system (Figure 3e,f (i)). These synchronized calcium signals are observed only in the multicellular system, but never in experiments of random culture (*n* = 4 experiments per group, Videos S1 and S2, Supporting Information). We calculate the synchrony of tissue units which is defined as cross‐correlation coefficient at zero lag time.^[^ ^26^ ^]^ The synchrony matrix shows a high degree of synchronisation in a subpopulation of closely packed tissue units (note the yellow region in the matrix of Figure 3e (ii)), while an evident lower synchrony is found in the random culture (Figure 3f (ii)). The results demonstrate that the multicellular morphology can be engineered to reproduce multicellular crosstalk and to develop populational‐level functions.

### 2.3 Morphology of the DRG Multicellular System and its Self‐Organisation

**Figure 4:**
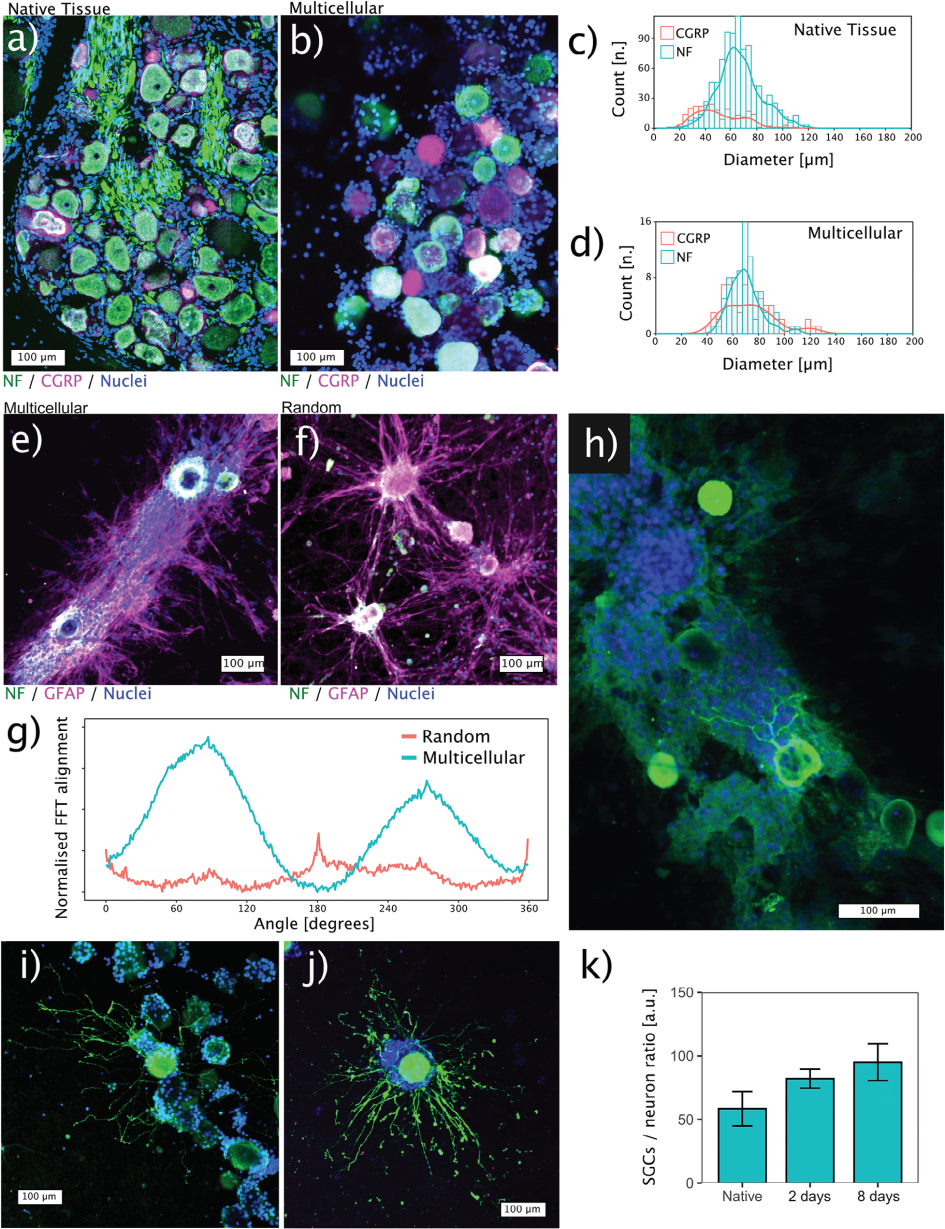
Morphology of the assembled multicellular system. Immunofluorescence staining of the native tissue a) and the assembled multicellular system b) closely recapitulates the tight cell‐to‐cell contact of the native DRG. Soma diameters are comparable between the multicellular assembly c) and the native bovine DRG tissue d). The multicellular assembly shows anisotropic cell self‐organisation e), whereas in the random culture, the self‐organisation of satellite glial cells appears to be limited on day 8 f), as evidenced by the quantification using 2D‐Fast Fourier transformation (FFT) alignment g). The self‐organised structure provides a guidance for axonal growth h). Axons sprouting in the collagen matrix in both the multicellular system i) and the random culture j) after 2 days of culture. k) The counting ratios of glia to neurons in the multicellular system at day 2 and day 8 are compared with native tissue. Data is presented as mean ± standard error in the bar plot. *n* = 3 replicates per group.

Neurons are densely packed in native DRG tissue (**Figure** **4**a). This close cell‐to‐cell proximity is successfully recapitulated in the hydrodynamic force‐assembled multicellular system (Figure 4b). The neuron sizes in the assembled multicellular system approximate those of bovine DRG native tissue (Figure 4c,d).^[^ ^8^ ^]^

Native DRG tissue is also known to have oriented cellular anisotropy. Figure 4a shows that the SGC nuclei are oriented in the same direction of axon fibers. We show that the ability of cells to self‐organize into oriented architecture is not limited to stem cells. Mature DRG cells also exhibit the ability to self‐organize. At day 8, SGCs in the multicellular system orient themselves along a precise direction connecting each other in an organized structure (Figure 4e). Importantly, in the random culture, SGCs display only a small trend to self‐organize. Merely few SGCs tend to extend and connect isolated DRG tissue units (Figure 4f). This is further confirmed by the 2D‐Fast Fourier Transform (FFT) analysis. The orientation distribution plot shows a homogeneous SGC alignment in the multicellular system, while the orientation distribution of non‐assembled control is mostly random (Figure 4g). Such anisotropic SGCs organization seems to guide and support axonal growth (Figure 4h), similar to what is observed in the native DRG tissue (Figure 4a).

Next, to study the sole effect of AF explant on nerve ingrowth and avoid the interference of the self‐organizing SGCs, we use the 2‐day culture to evaluate the ingrowth‐associated axonal sprouting. At day 2, the SGCs are still symmetrically enveloping the neuronal soma without any oriented spreading. Axons extend in the collagen matrix without the influence from SGCs (Figure 4i,j). The counting ratios of SGCs to neurons are 82:1 and 95:1 for day 2 and day 8 in the multicellular system which are not significantly different from the native tissue ratio. (Figure 4k) (*p* > 0.1 for all pairwise comparisons using 2‐sided Wilcoxon rank sum exact test, *n* = 3.)

### 2.4 The Neurotrophic (Growth‐Promoting) Effect of Cytokine‐Primed AF Depends on the AF‐Neuron Distance

**Figure 5:**
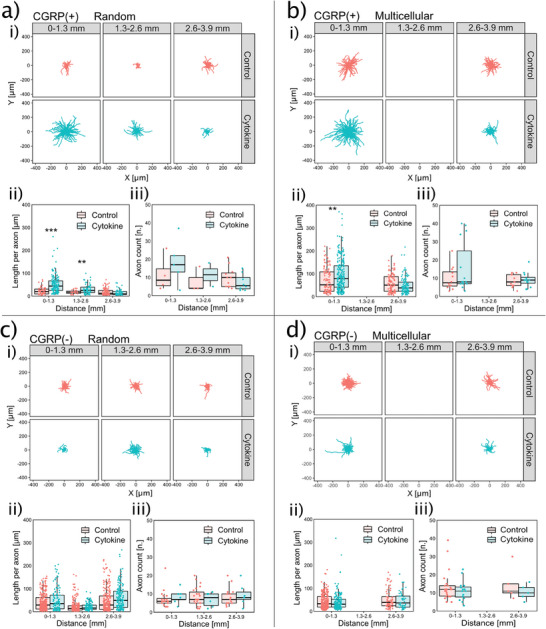
Effect of cytokine‐primed AF explant on axonal length and count depends on the AF‐neuron distance. Axonal morphologies are shown as a function of the distance from the AF explant in a–d (i). CGRP(+) axons in both random culture a) and multicellular system b) show a distance‐dependent increased length (ii) and count (iii) when exposed to a cytokine‐primed AF explant. CGRP(‐) axons do not show any distance‐dependent difference in length (i) or count (ii) in either random culture c) nor multicellular system d). Data is presented as boxplot which presents the interquartile range of data distribution. **: *p* < 0.01, ***: *p* < 0.001 by 2‐sided Wilcoxon signed‐rank test. *n* = 445, 647, 1150, and 700 axons in (ii) of (a–d), respectively. *n* = 51, 61, 85, and 48 neurons in (iii) of (a–d), respectively.

Axonal sprouting of neurons is influenced by both chemical cues from soluble factors and physical cues from cell adhesion and surface topography.^[^ ^27^ ^]^ Our goal is to exclusively investigate the chemical cues (*i.e*., the neurotrophic and neurotropic effects). The interference of physical cues may influence IVD nerve ingrowth in real situation but must be excluded when our research question is the sole effects of chemical cues. Indeed, a cytokine priming of the AF explant disrupts the anisotropic alignment of AF collagen extracellular matrix. (Figure S3, Supporting Information) Our model is designed so that axonal sprouting does not occur in the extracellular matrix of the AF, but in a collagen matrix‐based hydrogel. Immunofluorescence staining of the collagen fibers in the hydrogel provides evidence for their random orientation, which is not influenced by the sound assembly. (Figure S4, Supporting Information)

in vivo, the DRG neuronal soma is physically separated from IVD. We next investigate whether the distance between neuron soma and AF influences their communication in the co‐culture system. We prove the neurotrophic effect of the cytokine‐primed AF on the CGRP(+) neurons when the AF‐neuron distance is smaller than 1300 µm (AF‐close neurons). For AF‐close and CGRP(+) neurons, the average axonal length of cytokine‐primed AF group is 25 µm longer in the random culture (*p* < 0.001 by 2‐sided Wilcoxon signed‐rank test, *n* = 445 axons) (**Figure** **5**a (i,ii)), and 27 µm longer in the multicellular system (*p* = 0.0013 by 2‐sided Wilcoxon signed‐rank test, *n* = 647 axons) (Figure 5b (i,ii)) compared to the non‐cytokine control.

We find this neurotrophic effect of cytokine‐primed AF depends on the AF‐neuron distance. A closer AF‐neuron distance is associated with a longer axonal extension, thus displaying a negative correlation between the AF‐neuron distance and the length of CGRP(+) axons. The correlation coefficient (ρ) between the AF‐neuron distance and CGRP(+) axon length is ‐0.55 (*p* < 0.001 by Spearman's rank correlation coefficient test, *n* = 264 axons) in the cytokine‐primed AF group (Figure 5a (ii green)). The close AF‐neuron distance is also correlated with a higher count of CGRP(+) axons (axonal branch number) exhibiting a *ρ* value of ‐0.52 (*p* = 0.03 by Spearman's rank correlation coefficient test, *n* = 15 neurons) (Figure 5a (iii green)). The AF‐far neurons are less influenced by the cytokine‐primed AF explant and can be used as a control. When AF is not primed with cytokines, the axonal length and count of AF‐close neurons are not different from those of AF‐far neurons. The *ρ* value between AF‐neuron distance and CGRP(+) axonal length is ‐0.16 (*p* = 0.03 by Spearman's rank correlation coefficient test, *n* = 181 axons). The *ρ* value between AF‐neuron distance and CGRP(+) axonal count is 0.08 (*p* = 0.75 by Spearman's rank correlation coefficient test, *n* = 18 neurons) (Figure 5a,b (i–iii red)). Thus, there is no detectable neurotrophic effect in the non‐cytokine control.

These results point to a need to control the AF‐neuron distance. The hydrodynamic forces assemble a larger number of neurons to be AF‐close and potentially increases the sample size of neurons for analysis. This is of practical importance. In the random culture, many experiments without any neurons close to the AF explant have to be excluded. After performing the random culture 96 times using DRG from 12 cattle, only 9 CGRP(+) neurons with 186 axons are obtained in the AF‐close region. Instead, the assembled system achieves a sample size of CGRP(+) neuron more than 2‐fold higher than the random culture in the AF‐close region with only 8 experiments using DRG from 4 cattle. In the assembled multicellular system that is AF‐close, 27 CGRP(+) neurons with 355 axons are available for analysis (Figure 5a,b (ii,iii) number of data points).

For CGRP(‐) neurons, the AF explant is not showing any significant neurotrophic effect. The CGRP(‐) axon length and count are not different comparing AF‐close and AF‐far neurons and are not influenced by the cytokine‐priming of AF explant (Figure 5c,d (i–iii)). This agrees with the in vivo findings where axons growing into pain‐generating IVD are usually labelled by CGRP or SP.^[^ ^6^, ^9^ ^]^ CGRP is a specific marker of peptidergic nociceptors which is shared between species.^[^ ^28^ ^]^ Those CGRP(‐) / SP(‐) and neurofilament (NF)(+) neurons are regarded as low‐threshold mechanoreceptors and proprioceptors that sense light touch and body position.^[^ ^28^ ^]^ Based on the results of our model, the neurotrophic effect of cytokine‐primed AF is nociceptor specific.

### 2.5 The Neurotropic (Guidance) Effect of Cytokine‐Primed AF

**Figure 6:**
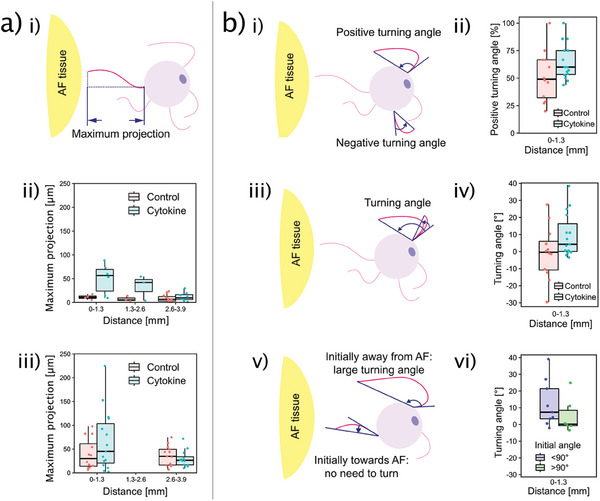
Effect of the cytokine‐primed AF explant on CGRP (+) axonal guidance. a) Schematic depiction illustrating the “maximum projection length” (i). Axons show a distance‐dependent “maximum projection length” in both random culture (ii) and multicellular system (iii). b) Turning angle of axons. (i) “Positive” and “negative” turning angles. (ii) A higher proportion of axons per neuron turn toward the cytokine‐primed AF explant, but not toward the non‐cytokine‐primed AF. iii) Illustration showing that larger turning angle corresponds to a larger AF guidance. (iv) Cytokine‐primed AF explant induces a larger turning angle than the control. As illustrated in (v) and quantitatively shown in (vi), the guidance on axons results in a larger turning angle only when axons initially sprout in the opposite direction in respect to AF (initial angle < 90°). Data is presented as boxplot which presents the interquartile range of data distribution. *n* = 27 neurons in (ii and iv) of (b). *n* = 15 neurons in (b‐vi).

To evaluate how far the CGRP(+) axon can grow toward AF, we projected the axon trajectory to the axis connecting AF and neuron cell body to measure the projection length. The maximum projection length of axons per neuron is evaluated (**Figure** **6**a (i)). A larger maximum projection length indicates that the axons extend more toward the AF. Results imply that the AF's influence on maximum projection length also depends on the AF‐neuron distance. In the cytokine‐primed AF group, the *ρ* values are ‐0.59 (*p* = 0.013, *n* = 17 neurons) and ‐0.25 (*p* = 0.21, *n* = 28 neurons) for random culture and multicellular system, respectively (Spearman's rank correlation coefficient test) (Figure 6a (ii,iii green)). However, in the non‐cytokine control, such correlation is not observed. The *ρ* values are 0.15 (*p* = 0.57, *n* = 18 neurons) and 0.02 (*p* = 0.92, *n* = 29 neurons) for random culture and multicellular system, respectively (Spearman's rank correlation coefficient test) (Figure 6a (ii,iii red)). These results indicate that the cytokine‐primed AF may have a neurotropic effect on CGRP(+) and AF‐close axons.

The developing nervous system is far more plastic (capability to change) than the adult, so embryonic and neonatal neuron culture are more commonly used to investigate how axons sense the environmental cues and decide the growing direction.^[^ ^29^ ^]^ Here, we evaluate the neurotropic effect of AF on mature neurons which has seldomly been characterised before. Since the random culture did not have enough neurons (9 AF‐close neurons), this analysis was only possible for the multicellular system (27 AF‐close neurons). The turning angle is a common indicator of axonal guidance.^[^ ^29^, ^30^ ^]^ It is defined by the difference between the initial growth angle and the ending growth angle (Figure 6b (i, iii, v)). An AF's guidance on attracting axon will result in a positive turning angle, while an axon turning away from the AF explant is represented as a negative turning angle (Figure 6b (i)). In the non‐cytokine control, the proportion of positive turning axons per neuron averages at 50% suggesting equal numbers of axons turning toward and away from AF. Thus, there is no guidance from the AF tissue explant (Figure 6b (ii red)). In contrast, in the cytokine‐primed AF group, 60% of CGRP(+) axons per neuron have a positive turning angle. (*p* = 0.12 comparing cytokine to control by 2‐sided 2 sample *t* test, n = 27 neurons) Notably, in the cytokine‐primed AF group, 12 out of 15 CGRP(+) neurons (80%) have majority of their axons turning with a positive angle (Figure 6b (ii green)). (*p* = 0.04 by Pearson's Chi‐squared test, *n* = 27 neurons)

The turning angle value is averaged to be ‐0.4° for non‐cytokine control but is 4.3° for the cytokine‐primed AF group (Figure 6b (iv)) (*p* = 0.1 by 2‐sided Wilcoxon signed‐rank test, n = 27 neurons). The axonal guidance is represented by a larger turning angle only when the axon is initially pointing away from the AF (Figure 6b (iii)). Nevertheless, when the axons initiated their growth already toward the AF, the AF's guidance does not need a turning, but instead to guide the axons to persist the initially “correct” direction (Figure 6b (v)). Considering this, we divide the data points of cytokine‐primed AF group into initial angle greater than 90° and smaller than 90°. We do find a larger turning angle when the axons are initially pointing away from the AF (initial angle < 90°) compared to axons initially already pointing toward the AF (initial angle > 90°) (Figure 6b (vi)) (*p* = 0.22 by 2‐sided Wilcoxon signed‐rank test, *n* = 15 neurons). Taking together, the cytokine‐primed AF shows an ingrowth‐related axonal guidance.

### 2.6 The AF's Neurotrophic Effect is Mediated by Viable AF

**Figure 7:**
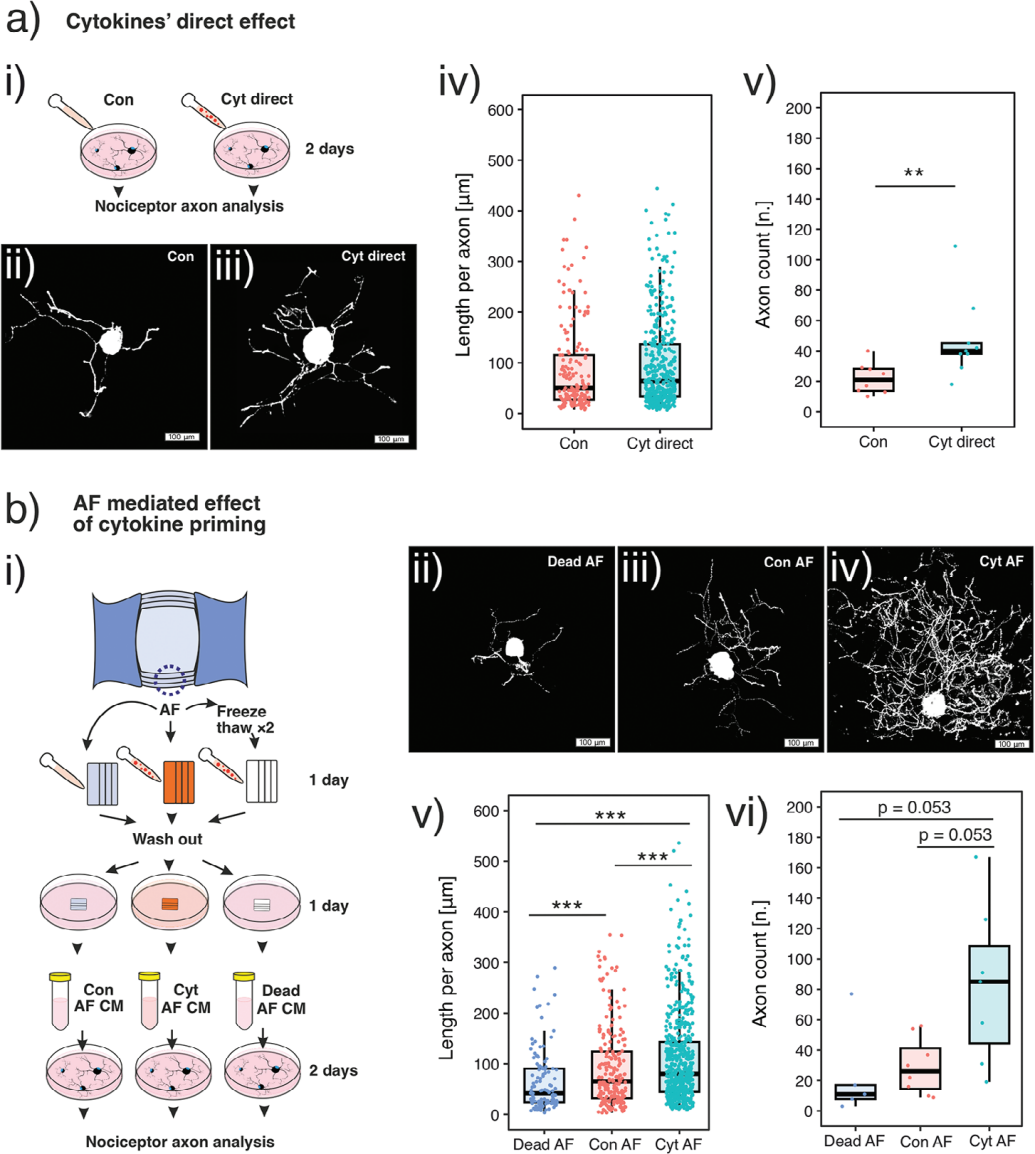
Neurotrophic effect of AF explant is mediated by viable AF explant. Schematics of the study design are presented in (a‐i) and (b‐i), respectively. a) Direct neurotrophic effect of cytokines. Cytokines directly increase CGRP(+) axon length and frequency. b) Neurotrophic effect of cytokine‐primed AF. Viable AF primed by cytokines (Cyt AF) increases CGRP(+) axon length and frequency (i.e., the neurotrophic effect) compared to non‐primed AF (Con AF). Dead AF's conditioned medium (Dead AF) shows no neurotrophic effect, although they are processed by the same cytokine priming. This excludes the possibility that the unwashed cytokines confound the result. Data is presented as boxplot which presents the interquartile range of data distribution. In (a‐v), **: *p* < 0.01 by 2‐sided Wilcoxon signed‐rank test, *n* = 17 neurons. In (b‐v), ***: *p* < 0.001 by 2‐sided Wilcoxon signed‐rank test, *n* = 927 axons. In (b‐vi), *p* value is calculated using 2‐sided Wilcoxon signed‐rank test, *n* = 20 neurons.

To consolidate the neurotrophic effect of the cytokine‐primed AF, we need to rule out the confounding effect of leftover cytokines after AF washout. Direct application of these cytokines to primary bovine DRG cells increases the length and frequency of CGRP(+) axons by 28.3% (*p* = 0.82 by 2‐sided Wilcoxon signed‐rank test, *n* = 296 axons) and 142.6% (*p* = 0.0044 by 2‐sided Wilcoxon signed‐rank test, *n* = 17 neurons), respectively (**Figure** **7**a). This indicates that these cytokines have a direct neurotrophic effect on nociceptors. Although we wash the AF after cytokine priming, some residual cytokines may be transferred to the DRG cells. The next study is designed to show that the neurotrophic effect comes from the viable AF and not from the residual cytokines added to prime the AF.

This is achieved by adding a control group in which we prime the dead AF explant (AF processed by 2 freeze‐thaw cycles) with cytokines. After the same washout as in the coculture system, we use the AF's conditioned medium (CM) to stimulate DRG cells. In the other 2 experimental groups, CMs of viable AF primed with and without cytokines are applied to DRG cells. We find that the CM of viable AF primed with cytokines increases the length and count of CGRP(+) axons by 22% (*p* < 0.001 by 2‐sided Wilcoxon signed‐rank test, *n* = 927 axons) and by 2.2‐fold (*p* = 0.053 by 2‐sided Wilcoxon signed‐rank test, *n* = 20 neurons) compared to the CM of viable AF not primed with cytokines (Figure 7b). Nevertheless, the dead AF does not increase the length or count of nociceptor axons despite being processed by the same cytokine priming (Figure 7b). The neurotrophic effect of the cytokine‐primed AF cannot be explained by the residual cytokines because the dead AF does not show the same neurotrophic effect after the same cytokine priming and washout.

### 2.7 Cytokine Primed AF Dysregulate a Broad Range of Gene Expression in DRG Cells

**Figure 8:**
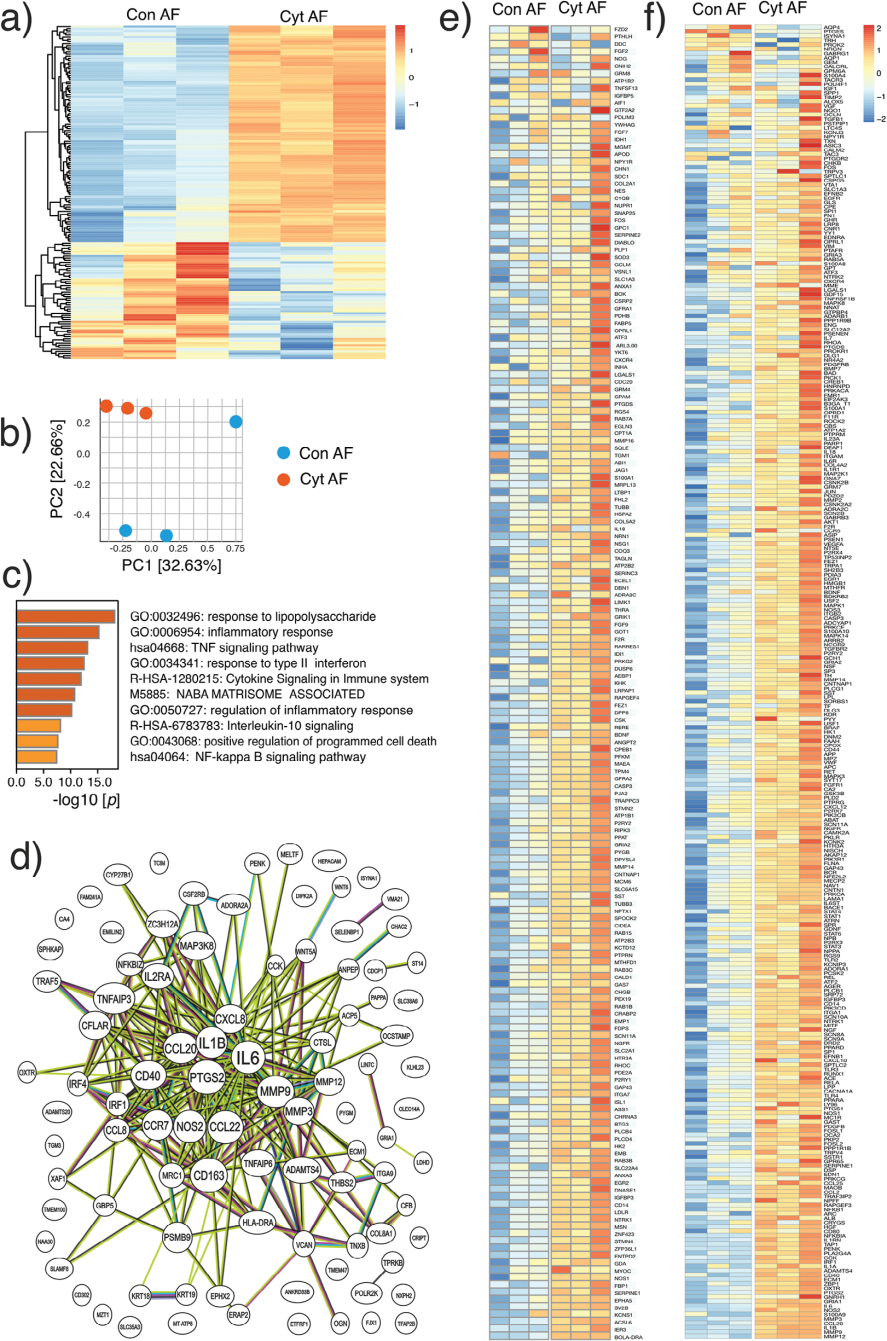
Bulk RNA sequencing of bovine DRG cells. The effect of cytokine‐primed AF (Cyt AF) is compared to non‐primed AF (Con AF). a) Heatmap summarising the differentially expressed genes (DEGs). b) Principal component analysis (PCA) showing the separation of Cyt AF and Con AF groups. c) The top regulated GO terms determined by Metascape. d) Protein‐protein interaction analysis of DEGs. e,f) The expression levels of genes associated with neurite outgrowth e) and pain f).

We performed bulk RNA‐seq for primary bovine DRG cells. DRG cells treated with cytokine‐primed AF CM are compared to those treated with non‐primed AF CM. We identified 157 differentially expressed genes (DEGs). Of these, 102 DEGs are over‐expressed; 55 are downregulated. (**Figure** **8**a) Principal component analysis (PCA) of genes higher than 1 transcript per million (TPM) shows different clusters between groups. (Figure 8b) We use Metascape (http://metascape.org) to identify genes with shared functions. We find DEGs enriched in Gene Ontology (GO) terms associated with inflammation. (Figure 8c) The network of these DEGs is predicted based on the literature text mining and co‐expression. (Figure 8d) Key hub genes in the network include IL6, IL1B, NFKBIZ, MAP3K8, PTGS2, TNFAIPs, MMPs and CCLs, *etc*. Next, we specifically investigated genes reported in previous literature that are associated with neurite outgrowth^[^ ^31^ ^]^ and pain.^[^ ^32^ ^]^ We find that the majority of these genes are upregulated in DRG cells treated with cytokine‐primed AF CM as compared to non‐primed AF CM. (Figure 8e,f)

### 2.8 Cytokine Primed AF's Effect on Calcium Signalling of DRG Neurons

**Figure 9:**
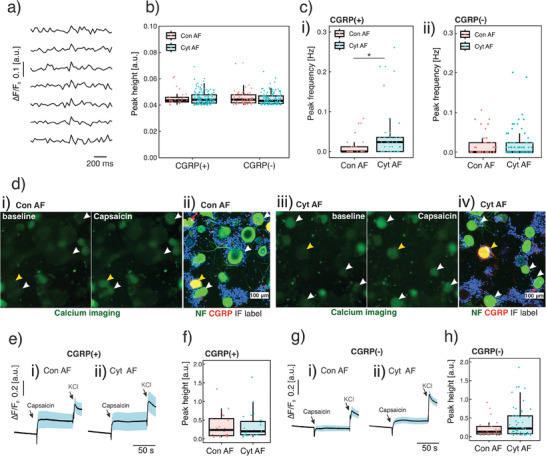
Calcium signals of bovine DRG neurons. CGRP(+) and CGRP(‐) subtypes of neurons are labelled using immunofluorescence following calcium imaging. The effect of cytokine‐primed AF conditioned medium (CM) (Cyt AF) is compared with non‐primed AF CM (Con AF). To exclude dead neurons in the analysis, neurons without a response to potassium chloride (KCl) are excluded. a) Spontaneous calcium transients are represented as normalised fluorescent peaks. b) The spontaneous calcium peak height is consistent comparing different neuronal subtypes and treatment groups. c) The cytokine‐primed AF increases the frequency of spontaneous calcium response in CGRP(+) neurons (i), but not in CGRP(‐) neurons (ii). d) The capsaicin‐induced (100 nm) calcium response in both CGRP(+) and CGRP(‐) neurons. e,f) The capsaicin‐induced calcium response is not different in CGRP(+) nociceptors between Con AF (e‐i) and Cyt AF (e‐ii) groups. g,h) Cytokine‐primed AF CM (g‐i) increases capsaicin induced calcium response in CGRP(‐) neurons compared to the non‐primed AF CM (g‐ii). e) and g) are mean normalised fluorescent curves among different neurons. Blue straps are their standard deviation. Data is presented as boxplot which presents the interquartile range of data distribution. For (c‐i), *: *p* < 0.05 by 2‐sided Wilcoxon rank sum test; *n* = 90 CGRP(+) neurons and *n* = 161 CGRP(‐) neurons.

Calcium imaging is well‐established in studying DRG neuron sensitisation associated with pain.^[^ ^33^ ^]^ Here, we investigate both spontaneous and capsaicin induced calcium signalling in CGRP(+) and CGRP (‐) neurons. This is achieved by an immunofluorescence labelling of CGRP and NF after calcium imaging. Spontaneous calcium signals are recorded in the peripheral region of neuronal soma and appear as short peaks in the ΔF / F_0_ waves. (**Figure** **9**a) These spontaneous calcium signals, the calcium flicker,^[^ ^33a^ ^]^ are associated with ectopic neuronal discharge^[^ ^34^ ^]^ and are responsible for pain‐related neurotransmitter release^[^ ^33a^ ^]^. We found that the amplitudes of the spontaneous calcium flicker are not different in different subtypes of neurons (CGRP(+) versus CGRP (‐)) and in different treatment groups (Con AF versus Cyt AF). (Figure 9b) Nevertheless, the peak frequency of calcium flicker in CGRP(+) nociceptors is increased from 0 to 0.024 Hz comparing the cytokine‐primed AF group to the non‐primed AF group (*p* = 0.04 by 2‐sided Wilcoxon rank sum test, *n* = 90 neurons). (Figure 9c‐i) The peak frequency in CGRP (‐) neurons is not different comparing different treatment groups (*p* = 1 by 2‐sided Wilcoxon rank sum test, *n* = 161 neurons). (Figure 9c‐ii) The results suggest that the spontaneous calcium signal is upregulated by cytokine‐primed AF in CGRP(+) nociceptors.

The transient receptor potential cation channel, subtype V, type 1 (TRPV1) which regulates calcium influx in neurons, plays a pivotal role in nociception and pain.^[^ ^35^ ^]^ The functions of TRPV1 can be investigated by applying TRPV1 agonist capsaicin to the DRG culture and evaluating the response of neurons under calcium imaging. We examine the capsaicin‐induced response in both CGRP(+) and CGRP(‐) neurons. (Figure 9d) The capsaicin‐induced calcium peak heights in CGRP(+) neurons is not different between groups. (Figure 9e,f) However, capsaicin‐induced calcium peak height in CGRP(‐) neurons is increased by 60% (*p* = 0.159 by 2‐sided Wilcoxon rank sum test, *n* = 161 neurons) (Figure 9g,h) indicating an increased nociceptive response.

## 3 Discussion

Chronic low back pain (LBP) is affecting more than 500 million people and is the leading cause of disability worldwide.^[^ ^36^ ^]^ Although most structures in the lumber region can contribute to LBP, the dysfunctional intervertebral disc (IVD) is recognised as the source of pain in 40% of LBP patients.^[^ ^37^ ^]^ The associated sensory nerve ingrowth in IVD may contribute to chronic pain.^[^ ^6c^ ^]^ The influence of IVD, particularly the AF, on axonal sprouting is mostly unveiled. Although IVD nerve ingrowth has been observed in vivo,^[^ ^9^ ^]^ it is influenced by many different factors. For example, a breakdown of AF matrix, a descending modulation from central nervous system, or an elevated growth factors in the circulation may all contribute to IVD nerve ingrowth. Due to the complexity of in vivo environment, it is difficult to extract and solely focus on a specific mechanism of nerve ingrowth. Here, we present a strategy of using an in vitro co‐culture model. Using this strategy, it is for the first time that we successfully demonstrated the AF's neurotrophic and neurotropic effects with confounding factors easily controlled in an in vitro setup.

Most in vitro study of DRG neurons are based on primary monolayer culture and cell line culture. Neurons are cultured on stiff plastic and glass surface and are subject to a different mechanical environment comparing to the in vivo situation.^[^ ^38^ ^]^ Culturing DRG neurons in extracellular matrix‐based hydrogel is more physiological,^[^ ^38a^ ^]^ but cells are still isolated, losing the multicellular architecture of native tissue. Culturing tissue explant *ex vivo* preserves the tissue architecture.^[^ ^39^ ^]^ Explant culture of neonatal^[^ ^40^ ^]^ and adult^[^ ^41^ ^]^ rodent DRG has been reported. Also, we have formerly reported the methodology of adult rabbit DRG explant culture.^[^ ^42^ ^]^ However, large animal or human DRGs are much larger than rodent and rabbit DRG, and the extracellular matrix is denser, preventing nutrients^[^ ^8^ ^]^ and oxygen diffusion.^[^ ^43^ ^]^ The culture of large animal or human DRGs in the form of tissue explant is challenging and has not been reported.

Our approach can overcome these limitations. Large animal DRGs are first disintegrated into micro‐scale tissue units, and then re‐assembled using mild acoustic hydrodynamic forces. In this way, DRG are maintained in a multicellular system with a controlled scale to reduce the risk of necrotic core. In our DRG multicellular system, the dimension of the assembled system can be controlled in a width of 300–500 µm (Figure 2; Figure S2, Supporting Information) and a thickness of 100–150 µm. The physiological culture condition of our multicellular system is evidenced by the high cell viability.

Former study showed that acoustic assembled embryonic mouse cortical brain cells exhibit inter‐neuron synchronization.^[^ ^21b^ ^]^ Little is known whether this shape‐to‐function relationship also exist in adult mature neurons. Our model shows a cell‐building morphology cue in inter‐neuronal crosstalk in the case of mature neurons. The inter‐neuronal crosstalk in DRG has been increasingly recognized as an essential mechanism of chronic pain^[^ ^16a^ ^]^. This knowledge may contribute to the improvement of the general DRG culture protocol, where the neural cells can be investigated in a populational level considering cell‐to‐cell communication and network.

Over decades, embryonic and neo‐natal DRG from chicken, mice and rat has greatly contributed to the understanding of sensory neural biology. However, the increasing call for translation from benchside to bedside requires models with higher clinical relevance. These lower vertebrates (e.g., rodents) are biologically different from human^[^ ^44^ ^]^. Also, the developing (embryonic and neonatal) nervous system is different from their adult and mature counterpart.^[^ ^45^ ^]^ Likewise, the maturity of neuron‐like cells derived from human induced pluripotent stem cells is not yet fully validated, and little is known about their similarities to mature native DRG neurons.^[^ ^13b^ ^]^ Experiments using adult human DRG are constrained by the limited availability and ethical considerations. Recently, researchers are beginning to focus on adult large animal DRG to perform transcriptome and to map genomic loci associated with human pain disease.^[^ ^28^ ^]^ Here, we obtain our adult large animal tissues from the abattoir. This provides a cheap and unlimited resource for research and development. More importantly, it does not involve the deliberate sacrifice of animals. Much work is needed to understand whether bovine tissue is more relevant to humans than lower vertebrates. Indeed, the diameter of the bovine DRG neuron is 20–120 µm (Figure 4c,d), which is closer to previously reported human DRG neurons (20–100 µm) compared to rodents (20–50 µm).^[^ ^8^ ^]^ We are aware that a comprehensive characterization of the bovine DRG phenotype compared to human DRG is needed to prove the clinical relevance of the bovine tissue.

We used IL‐1β and TNF‐α to prime AF tissue in this model. These 2 cytokines are well known to be associated with clinical discogenic pain.^[^ ^46^ ^]^ Our previous study found that the cytokines upregulate the expression of multiple proinflammatory related genes in dissociated AF cell culture.^[^ ^47^ ^]^ Here, we investigate the role of IL‐1β and TNF‐α on the histology of AF tissue. Our findings show that these cytokines disturb the collagen fiber alignment in the AF explant, (Figure S3, Supporting Information) which agree with a former study that these cytokines are responsible for IVD extracellular matrix degradation.^[^ ^48^ ^]^

Despite much is already known about how these pro‐inflammatory cytokines influence IVD biology, it is the first time that the role of the cytokines on AF‐nerve communication is modelled in vitro. Our results agree with former in vivo study that a TNF‐α injection leads to a nociceptor nerve ingrowth.^[^ ^49^ ^]^ Also, IVD is exposed to other environmental stimuli such as injury^[^ ^50^ ^]^ and mechanical loadings^[^ ^51^ ^]^ *etc*. How the other environmental stimuli influence IVD nerve ingrowth in our model needs to be investigated in the future.

in vitro and in vivo models can be used to address different types of research questions. The purpose of in vitro models is not to recapitulate the real in vivo complexity, but rather to focus on specific mechanisms out of the complexity. This is easier to achieve with a controlled in vitro experimental setup. However, we recognize the importance of validating in vitro findings in vivo.

Although the present study focuses on IVD nerve ingrowth, altered sensory innervation is observed in other diseases, such as osteoarthritis^[^ ^52^ ^]^ and bone fracture non‐union^[^ ^5^ ^]^. In addition, sensory innervation is actively involved in inflammation and tissue repair^[^ ^1^, ^2^ ^]^ and may also regulate tumor progression.^[^ ^53^ ^]^ To date, the experimental manipulation and visualization of the tiny and complex axon structure hidden deep in vivo remain a challenge for the study of sensory innervation. Thus, our in vitro engineered multicellular system may help to overcome this challenge and pave the way for a broad application.

In summary, for the first time, the growth‐promoting (neurotrophic) and chemotactic (neurotropic) effects of AF on nociceptors are demonstrated. An in vitro model of nerve ingrowth using abattoir‐derived tissue provides a novel approach to animal replacement and potentially reduces the cost in research and development. Acoustic waves have been used to assemble multiple tissue types into a reproducible multicellular architecture, which has been shown to accelerate the physiologically relevant self‐organization.

## 4 Conclusion

We report the first in vitro model of AF nerve ingrowth using large adult animal tissue units. Mild hydrodynamic forces are used to spatially assemble DRG micro‐scale tissue units in a defined spatial organization. We prove the importance of recapitulating the in vivo morphology for developing physiological functions. By assembling DRG tissue units into a densely packed multicellular system, we achieved native‐like structural organization that led to multi‐level functional crosstalk (inter‐cellular and inter‐tissues). We demonstrate that the influence of cytokine‐primed AF explant on the extension and guidance of CGRP(+) axons depends largely on the AF‐neuron distance. This underpins the role of reproducing inter‐tissue/organ anatomical proximity when investigating their communications. This work proposes a new approach to biofabricate multi‐tissue/organ models for unravelling clinically relevant pathophysiology as well as discovering novel treatments.

## 5 Experimental Section

### AF Tissue Dissection, Culture, and Pro‐Inflammatory Cytokine Priming

Bovine tail IVDs were obtained from the local abattoir. Donor information (sex, age, and weight) is in Table S1 (Supporting Information). The IVDs were transected at cartilage growth plate, cleaned by a jet lavage and rinsed in phosphate buffered saline (PBS) containing 10% penicillin‐streptomycin (PS, 15140‐122, Gibco, UK). After removing the IVD endplates, the outer AF tissue was cut into small pieces with thickness ranging from 1 to 50 mm for AF viability essay (Figure S1, Supporting Information) and 1.5×1.5×1.5 mm cube for the co‐culture with DRG tissue units. The AF tissues were maintained in 4.5‐g L^−1^ glucose Dulbecco's Modified Eagle Medium (DMEM, 52100‐021, Gibco, Paisley, UK), 10% fetal calf serum (FCS, 35‐010‐CV, Corning, CA, US), 1% PS and 0.11‐g L^−1^ sodium pyruvate (P5280; Sigma‐Aldrich, JP) until use. The viability of the cultured AF tissue was evaluated by live dead staining. To allow dye penetration, the AF explants were pre‐processed by 1 mg mL^−1^ collagenase P (Roche, Mannheim, DE) for 1 h at 37°C. The dyes used for live dead staining were Calcein‐AM (1 µm, 17783, Sigma) and Ethidium homodimer‐1 (1 µm, 46043, Sigma). The AF explants were incubated in the dye for 30 min at 37°C. The imaging was performed using LSM 800 confocal (Zeiss, DE).

### Bovine DRG Micro‐Tissue Unit Preparation

Adult bovine DRGs were obtained from cervical spines at the local abattoir. Donor information is in Table S1 (Supporting Information). 10–12 DRGs per donor were disintegrated into micro tissue units using 6 mL collagenase P (4 mg mL^−1^, Roche, Mannheim, DE) in a 15 mL Falcon tube on an orbital shaker for 2 h at 37°C. A manual shaking of the digesting tissue was performed every 15 min. The digested compound went through a 100 µm cell strainer (Falcon, Corning, US) and centrifuged through 5 mL of PBS containing 15% BSA (centrifugation speed at a speed 600 g and duration for 7 min) in a 50 mL Falcon tube. The pellet was re‐suspended in 500 µL DMEM/F12 (50% v/v, DMEM from Gibco, 52100‐021, UK and F‐12 Ham from Sigma, N6760, UK) supplemented with 10% FCS, 20 mm HEPES (15630122, Thermo Fisher, US) and 1% ITS (354352, Sigma‐Aldrich, MO, USA). 50 µL of the re‐suspended solution was stained by equal amount of 0.4% trypan blue (T8154, Sigma) and counted in a haematocytometer.

### Collagen Matrix Preparation

Collagen solution containing DRG tissue units was prepared by sequential mixing of the following components: 10% of 10× concentrated DMEM/F12, 10% (v/v) FCS, 1% (v/v) ITS, 20 mM HEPES, 62% (v/v) deionised water, 0.5 mg mL^−1^ Collagen I (stock at 10 mg mL^−1^, cat.no. 354249, Corning, MA, US), and 10% (v/v) DRG tissue unit solution (1.2 × 10^5^ units mL^−1^). For each 100 µL stock collagen solution (10 mg mL^−1^), 6 µL 7.5% sodium bicarbonate was added to adjust the pH. This preparation was on ice and the SIM pattern was performed immediately following the solution preparation.

### Hydrodynamic Assembly of DRG Micro‐Tissue Units Around AF Explant

The AF explants were trimmed to cubes sized at 1.5×1.5×1.5 mm and were embedded in 360 µL of 2 mg mL^−1^ collagen in a culture frame. After 12 h, assembly was performed on top of the AF‐embedding collagen in a shallow cylindrical space with a diameter of 25 mm and height of 2 mm. The volume of the DRG containing collagen solution was 575 µL. The assembly protocol is based on a former publication.^[^ ^21a^ ^]^ Briefly, the excitation frequency was 60 Hz and amplitude 0.5 g. The duration of assembly was 2 min. Once collagen formed a hydrogel, the frames were moved to a 37°C incubator supplied with 90% humidity and 5% CO_2_.

### Immunofluorescence

For CGRP and NF labelling, the primary antibodies included rabbit anti‐CGRP antibody (1:500 dilution volume, cat.no. 24112, Immunostar, Sodiag Avegno, WI, US) and mouse anti‐neurofilament 200 antibodies (NF200, 1:50 dilution, cat.no. OMA1‐06117, Thermo scientific, NL). The primary antibody incubation was at 4°C for 24 h. After washes, this was followed by 2 h of secondary antibody incubation. The secondary antibodies included goat anti‐mouse AlexaFluor 488 conjugated antibody (1:100, A‐11029, Thermo Fisher, OR, US) and donkey anti‐rabbit AlexaFluor 680 (1:100, A32802, Thermo Fisher, OR, US). Nuclei were stained using Hoechst (2 µm, Sigma, cat. n. 14530, US). The gel volume was included for the calculation of antibody dilution. LSM‐800 confocal was used for imaging. Z‐stack was performed to ensure that the whole length of axons was covered in the images. For GFAP labelling of SGCs combined with neuron labelling (NF and TUBB3), the primary antibodies included rabbit anti‐GFAP polyclonal antibody (1:200, Dako, CA, USA) and mouse anti‐TUBB3 (1:50, MAB1637, Sigma, CA, USA). The staining and imaging method was the same as the labelling of CGRP and NF. The anisotropy of SCGs was characterised by the formerly reported 2D‐ Fast Fourier Transform (FFT) method.^[^ ^54^ ^]^ Briefly, the intensity profiles were obtained summing all the pixel fluorescent intensity (I) along a column of the image and normalising the intensity profile to 0.

### Outgrowth Analysis

The image segmentation and measurement were performed using imageJ Fiji (1.53q NIH, US) based on java 1.8.0_322 (64‐bit). Simple neurite tracer plugin (SNT)^[^ ^55^ ^]^ was used to segment axons in z‐projected 3D z‐stacked images. The segmented axons were saved in “swc” format and imported into “R studio” (1.1.383) based on “R” (3.6.2) using the “nat” package. Data arrangement and statistic were performed using “tidyverse” package within R. Visualisation of the data was performed using “ggplot2” package.

### Calcium Imaging

The calcium imaging protocol has been described formerly.^[^ ^56^ ^]^ Briefly, calcium imaging was performed using 5 µm Fluo‐4‐AM (ThermoFisher, F14201, US) in Krebs‐Ringer's solution containing NaCl 119 mm, KCl 2.5 mm, NaH_2_PO4 1.0 mm, CaCl_2_ 2.5 mM, MgCl_2_ 1.3 mm, HEPES 20 mm and D‐glucose 11.0 mm. Time‐lapse images were acquired using a LSM800 microscope with wide field epi‐fluorescent camera at 5 × magnification and 5 × 5 binning. The image acquisition rate was 30Hz. Calcium imaging videos were further analysed with ImageJ Fiji (1.53q NIH, US) based on java 1.8.0_322 (64‐bit). The synchrony between ROIs was evaluated following formerly reported methodology.^[^ ^26^ ^]^

To evaluate the neuronal sensitisation, the regions of interests (ROIs) were manually selected in the peripheral region of neuronal soma, and the fluorescence intensity was extracted from each ROI. The fluorescence intensity data was then imported into “R studio” for further analysis. The baseline fluorescence signal (F_0_) was defined as the average fluorescent intensity of the first 1000 time frames. The real‐time fluorescent intensity was normalised: ΔF / F_0_ = (F – F_0_) / F_0_. The spontaneous peaks of ΔF / F_0_ were detected based on the derivative of ΔF / F_0_ larger than 0.04. The peak height was calculated by subtracting ΔF / F_0_ by an average value of ΔF / F_0_ over a rolling time window (40 time points) around the peak event. A spontaneous calcium signal was defined based on a peak height larger than 0.04. This threshold value has been shown to successfully exclude noise based on our former calcium flickering evaluations in dead neurons.^[^ ^57^ ^]^ The capsaicin induced peak height was calculated by subtracting the maximum ΔF / F_0_ after adding capsaicin by an average value of ΔF / F_0_ over a time window (500 time frames) before capsaicin was added.

### Sample Preparation, RNA Purification, and Quality Assessment

Bulk RNA sequencing analysis was performed to compare DRG cells treated with cytokine‐primed AF CM versus DRG cells treated with non‐cytokine‐primed AF CM (i.e., Cyt AF versus Con AF). Two technical replicates from each group were included; a third replicate was obtained by pooling equimolar amounts from the first and second technical replicates. Total RNA was isolated using TRIzol™ Reagent (Invitrogen, Thermo Fisher Scientific Inc., Waltham, MA, USA or other) according to the manufacturer's instruction. RNA yield and quality were determined on a NanoDrop™ 2000 spectrophotometer (Thermo Fisher Scientifics Inc., Waltham, MA, USA). Total RNA concentration was measured using Qubit™ RNA BR Assay Kit on a Qubit™ 4.0 Fluorometer (Invitrogen, Thermo Fisher Scientific Inc.). The RNA integrity was assessed using an Agilent 4200 TapeStation System (G2991AA) with the Agilent High Sensitivity RNA ScreenTape Assay (Agilent Technologies Inc., Santa Clara, CA, USA). All RNA samples showed an RNA integrity number (RIN) from 7.6 to 8.3.

### mRNA‐Sequencing Library Construction

For mRNA‐seq library construction, 500 ng of total RNA per sample was captured using oligo (dT) magnetic beads‐based protocol using the Illumina^®^ Stranded mRNA Prep Ligation Kit (Illumina, San Diego, CA, USA). The kit allows to sequence the coding and noncoding transcriptomes that are polyadenylated with strand‐specific information. Libraries were prepared according to the manufacturer's instructions. The concentration of the final libraries was measured using Qubit™ 1 × dsDNA HS Assay Kit on a Qubit^®^ 4.0 Fluorometer (Invitrogen, Thermo Fisher Scientific Inc.) and their quality was assessed with Agilent 4200 TapeStation System and Agilent High Sensitivity D1000 ScreenTape Assay (Agilent Technologies Inc.). Libraries were then sequenced on an Illumina NextSeq™ 550 platform (Illumina Inc.) using a NextSeq 500/550 High Output Kit v2.5 (150 cycles, 2 × 75 bp read length, paired end) (Illumina Inc.) to achieve a sufficient read depth for the bioinformatics analysis.

### Bioinformatics Analysis

Read quality was verified using FastQC.^[^ ^58^ ^]^ Subsequently, reads were analysed using RSEM.^[^ ^59^ ^]^ STAR^[^ ^60^ ^]^ was used as an aligner. Bos_taurus.ARS‐UCD1.2 was used as the genome reference. The genome annotation file used in RSEM analysis was the Bos Taurus ARS‐UCD1.2 version 109. Both reference FASTA file and genome annotation file were downloaded from Ensembl database.^[^ ^61^ ^]^ Only genes with TPM > 1 in at least one sample were considered expressed and were included in the following analysis. PCA plot of expressed genes was produced using “prcomp” package in R. Differentially expressed genes (DEGs) were compared between DRG cells primed with cytokine treated AF CM versus those primed with non‐cytokine treated AF CM (Cyt AF vs Con AF) using DESeq2^[^ ^62^ ^]^ with |log_2_FC| > 0.5 and FDR < 0.1 as thresholds.

The functional analysis of DEGs was performed using Metascape.^[^ ^63^ ^]^ A protein‐protein interaction network of this list of genes was constructed using STRING.^[^ ^64^ ^]^ The list of genes associated with neurite outgrowth described by Szpara *et al.*,^[^ ^31^ ^]^ and the list of genes associated with pain described by Jamieson *et al.*,^[^ ^32^ ^]^ were converted to human orthologous genes. Lists of human – bovine orthologous genes and human – mouse orthologous genes were downloaded from Ensembl Biomart. The expressions of genes in these two lists were ranked and presented in heatmaps. Heatmaps report the log_2_FC sorted expression values. Heatmap figures are produced using “pheatmap” package in R. Figures are formatted using CorelDRAW (Graphics Suite 2021, ON, US) for ease of visualisation.

### Statistical Analysis

Statistical analysis was performed using “R studio” (1.1.383) based on “R” (3.6.2) using the “dplyr” package. For outgrowth analysis, 16 bovine donors were included in the study (donor information in Table S1). The neurons were subdivided based on their distance to the AF explant (0–1.3 mm, 1.3–2.6 mm, and 2.6–3.9 mm). If the count of neuron in a donor was less or equal to 1 at a certain distance range, this donor at this specific distance range was excluded for analysis. In the random culture, 445 axons from 51 CGRP(+) neurons and 1150 axons from 85 CGRP(‐) neurons were included for the analysis. In the multicellular system, 647 axons from 66 CGRP(+) neurons and 700 axons from 75 CGRP(‐) neurons were included. For other experiments, the sample size is described in results and figure legends.

Shapiro‐Wilk test was performed to evaluate the normality of data distribution (*p* > 0.1). Homogeneity of variances were evaluated using Bartlett test (*p* > 0.1). When the data was not normally distributed or when the variances were not considered equal between groups, the statistical significance was evaluated using 2‐sided Wilcoxon signed‐rank test. A pairwise 2‐sided Wilcoxon signed‐rank test was performed when 3 or more groups were included. The correlation between AF‐neuron distance and outgrowth measurements (length and count) was evaluated using Spearman's rank correlation coefficient test.

## Conflict of Interest

The authors declare no conflict of interest.

## Author Contributions

J.M. performed methodology, investigation, data curation, writing of original draft, reviewing & editing. J.E. performed investigation. S.G. performed writing, reviewing & editing. M.A. and T.S. performed conceptualization, methodology, funding acquisition, supervision, writing of orignal draft, reviewing & editing.

## Supporting information

Supporting Information

Supplemental Video 1

Supplemental Video 2

## Data Availability

The data that support the findings of this study are available from the corresponding author upon reasonable request.
