# Supplementary material for: Engineering Sensory Ganglion Multicellular System to Model Tissue Nerve Ingrowth
Source: Adv Sci (Weinh). 2023 Dec 19;11(11):2308478. doi: 10.1002/advs.202308478 (PMC10953573; doi:10.1002/advs.202308478)
Supplement: Supplementary file 1 — Supporting Information [file ADVS-11-2308478-s002.pdf]

ADVANCED SCIENCE
Open Access

Supporting Information

for *Adv. Sci.*, DOI 10.1002/advs.202308478

Engineering Sensory Ganglion Multicellular System to Model Tissue Nerve Ingrowth

*Junxuan Ma, Janick Eglauf, Sibylle Grad, Mauro Alini and Tiziano Serra**

Supporting Information

Engineering Sensory Ganglion Multicellular System to Model Tissue Nerve Ingrowth

Junxuan Ma, Janick Eglauf, Sibylle Grad, Mauro Alini, Tiziano Serra

J. Ma, J. Eglauf, S. Grad, M. Alini, T. Serra
AO Research Institute, Clavadelerstrasse 8, Davos, 7270, Switzerland

J. Eglauf
ETH Zürich, Rämistrasse 101, 8092, Zürich, Switzerland
T. Serra
Complex Tissue Regeneration Department, MERLN Institute for Technology-Inspired Regenerative Medicine, Maastricht University, Netherlands

E-mail: Tiziano.Serra@aofoundation.org

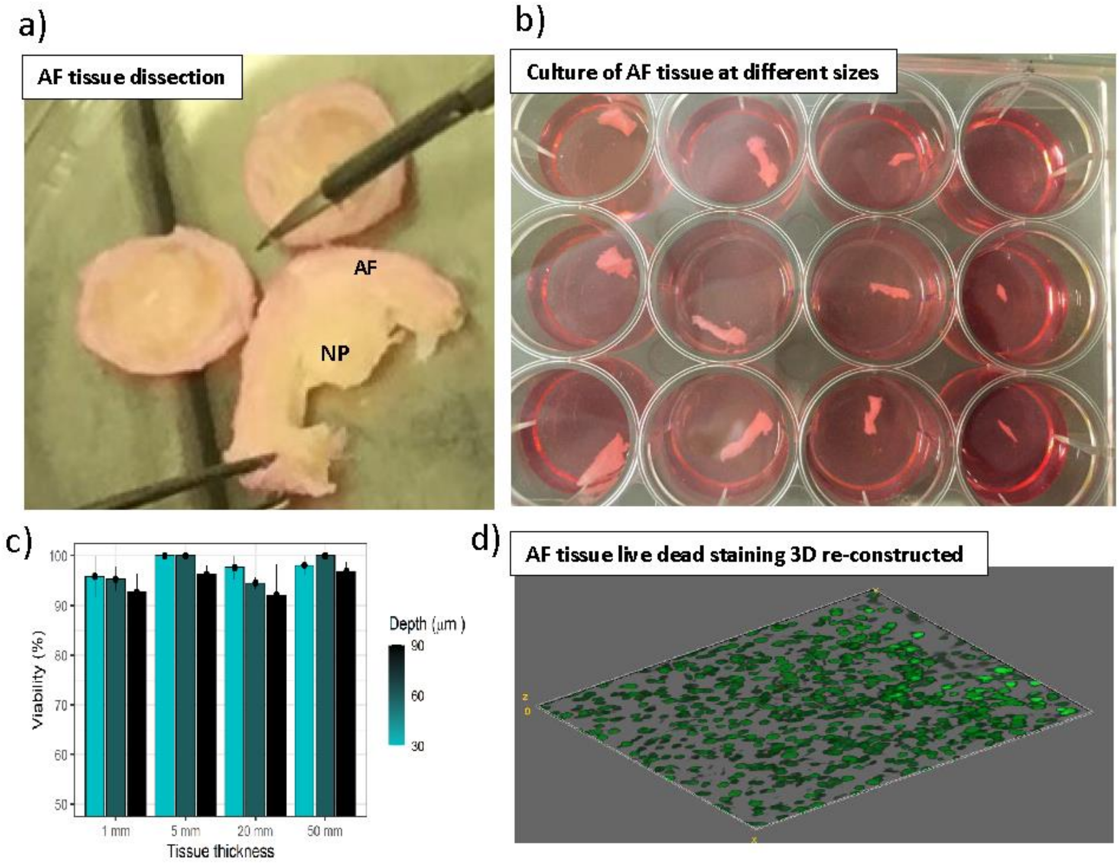

Figure S1. The culture of AF tissue explant. **a)** Dissection of AF tissue. **b)** Outer AF tissues are trimmed into different sizes for culture. **c)** The live dead staining of AF tissue after 9 days of culture shows a viability higher than 90% and this is not influenced by the trimming size of the explant. **d)** 3D reconstruction of confocal z-stack images of the live-dead-staining AF cells to 90 µm depth from the surface of AF explant.

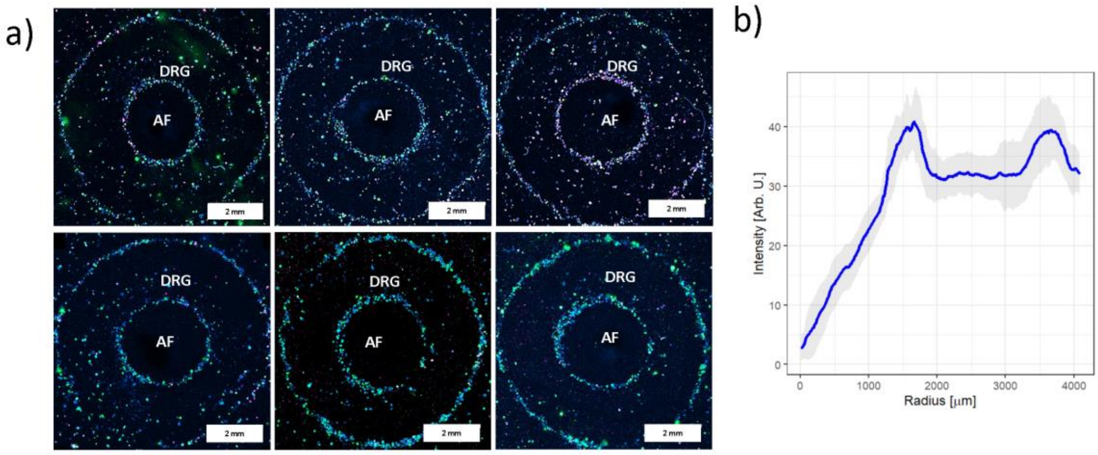

Figure S2. Geometry of the hydrodynamic patterning. **a)** Six experiments showing the consistency of the patterned DRG multicellular system. These are tiled immunofluorescence images labelling NF200 (green), CGRP (purple) and nuclei (blue). **b)** Radial profile of the patterned cellular geometry evaluated as in Di Marzio *et al.*[1] The 2 peaks represent the 2 rings, and the width of the peaks indicates the ring thickness. The x-axis location of the peaks shows diameter of the rings. Grey stratum represents standard deviation among different experiments.

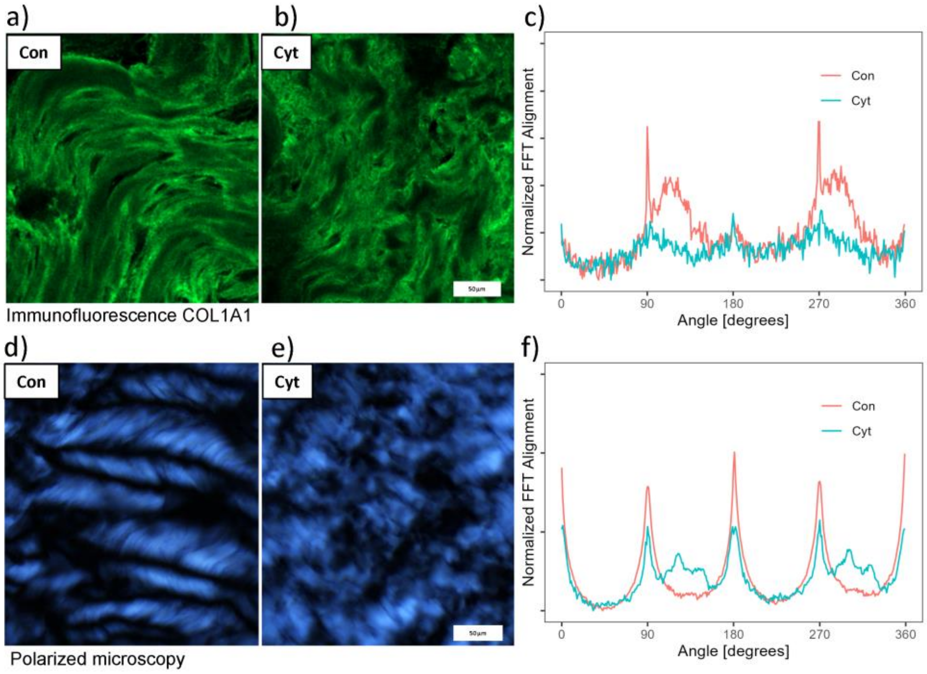

Figure S3. Effect of cytokines on extracellular matrix organisation of AF tissue. **a-b)** Immunofluorescence staining of collagen type Ⅰ (COL1A1) in the 10 µm thick cryosection of AF tissue without (Con) and with (Cyt) cytokine priming. **c)** Fast Fourier Transform (FFT) analysis of the collagen fibre alignment in the immunofluorescence images. **d-e)** Polarised light imaging in the cryosection of AF tissues. **f)** FFT analysis of the extracellular matrix alignment in the polarised light imaging.

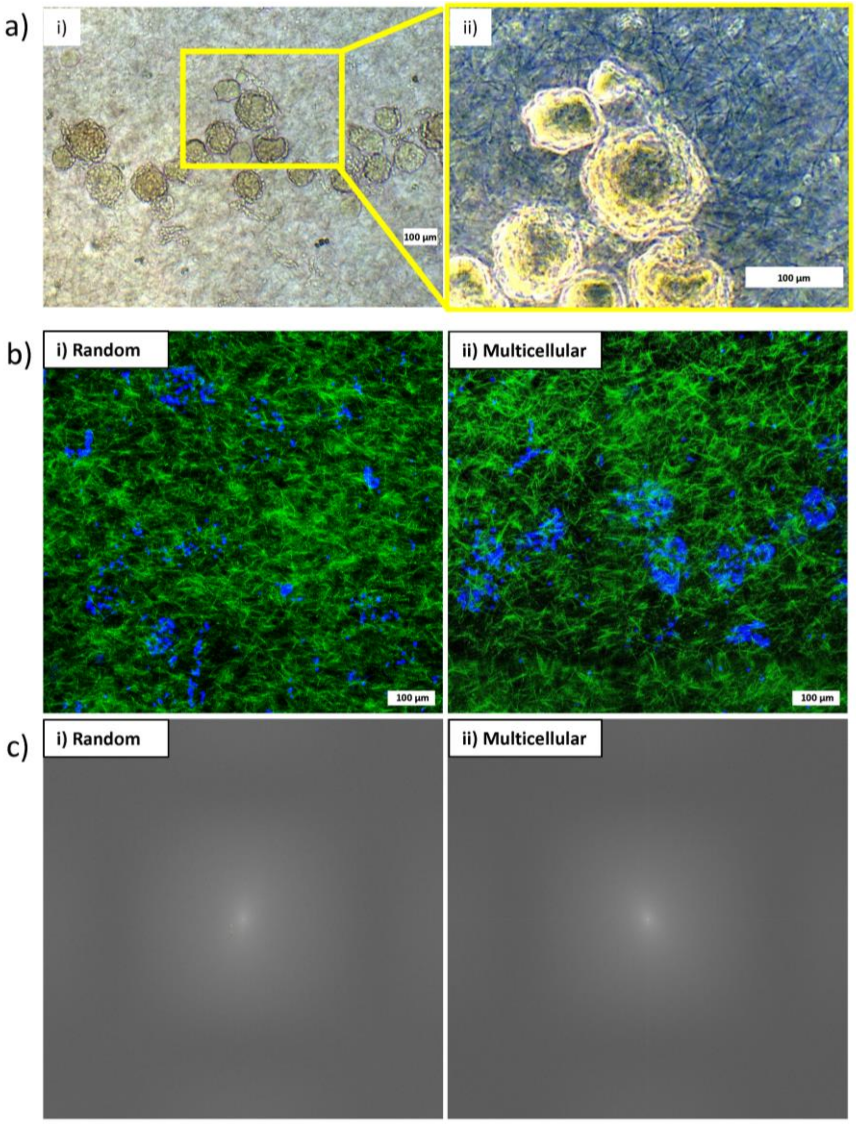

Figure S4. Collagen fibres in the collagen matrix-based hydrogel surrounding neurons. **a)** Phase contrast images of collagen surrounding the multicellular system. **b)** Immunofluorescence staining of collagen fibres around random and multicellular cells. Green channel is the collagen staining; blue channel is the DAPI staining of nuclei. **c)** Fast Fourier transformation of the collagen-stained images (green channels). No anisotropic orientation is observed.

Table S1. Donor information of bovine annulus fibrosus tissues (AF) and bovine dorsal root ganglion tissues (DRG)

Donor ID	Sex	Age (month)	Weight (kg)	Tissue Type
#1 Random[a)]	Female	12	200	AF
#2 Random	Male	10	190	AF
#3 Random	Male	10	190	AF
#4 Random	Male	10	190	AF
#5 Random	Male	6	148	AF
#6 Random	Male	12	200	AF
#7 Random	Male	12	192	AF
#8 Random	Male	5	136	AF
#9 Random	Male	10	235	AF
#10 Random	Male	10	167	AF
#11 Random	Female	10	125	AF
#12 Random	Male	11	240	DRG
#13 Random	Male	10	210	DRG
#14 Random	Male	12	204	DRG
#15 Random	Male	12	204	DRG
#16 Random	Male	10	167.4	DRG
#17 Random	Male	9	142.4	DRG
#17 Multicellular[b)]	Male	13	230	AF
#18 Multicellular	Male	12	235	AF
#19 Multicellular	Female	13	244	AF
#20 Multicellular	Female	8	180	AF
#21 Multicellular	Female	6	132	DRG
#22 Multicellular	Female	10	169.8	DRG

[a)] Random: the DRG tissue units seeded randomly around AF without assembling; [b)] sound-assembled DRG into multicellular system around AF.

References

[1] N. Di Marzio, P. Ananthanarayanan, A. G. Guex, M. Alini, C. Riganti, T. Serra, *Materials Today Bio* **2022**, *16*, 100357, https://doi.org/10.1016/j.mtbio.2022.100357.
